# Time-varying regularity of changes in biomechanical properties of the corneas after removal of anterior corneal tissue

**DOI:** 10.1186/s12938-021-00948-7

**Published:** 2021-11-20

**Authors:** Di Zhang, Xiao Qin, Haixia Zhang, Lin Li

**Affiliations:** 1 School of Biomedical Engineering, Capital Medical University, Beijing, 100069 China; 2 Beijing Key Laboratory of Fundamental Research on Biomechanics in Clinical Application, Capital Medical University, Beijing, 100069 China

**Keywords:** Corneal elastic modulus, Stress relaxation, Uniaxial tests, Corneal refractive surgery

## Abstract

**Background:**

The corneal biomechanical properties with the prolongation of time after corneal refractive surgery are important for providing a mechanical basis for the occurrence of clinical phenomena such as iatrogenic keratectasia and refractive regression. The aim of this study was to explore the changes of corneal elastic modulus, and stress relaxation properties from the 6-month follow-up observations of rabbits after a removal of anterior corneal tissue in simulation to corneal refractive surgery.

**Methods:**

The anterior corneal tissue, 6 mm in diameter and 30–50% of the original corneal thickness, the left eye of the rabbit was removed, and the right eye was kept as the control. The rabbits were normally raised and nursed for 6 months, during which corneal morphology data, and both of corneal hysteresis (CH) and corneal resistance factor (CRF) were gathered. Uniaxial tensile tests of corneal strips were performed at months 1, 3, and 6 from 7 animals, and corneal collagen fibrils were observed at months 1, 3, and 6 from 1 rabbit, respectively.

**Results:**

Compared with the control group, there were statistical differences in the curvature radius at week 2 and month 3, and both CH and CRF at months 1, 2, and 6 in experiment group; there were statistical differences in elastic modulus at 1, 3, and month 6, and stress relaxation degree at month 3 in experiment group. The differences in corneal elastic modulus, stress relaxation degree and the total number of collagen fibrils between experiment and control groups varied gradually with time, and showed significant changes at the 3rd month after the treatment.

**Conclusions:**

Corneas after a removal of anterior corneal tissue undergo dynamic changes in corneal morphology and biomechanical properties. The first 3 months after treatment could be a critical period. The variation of corneal biomechanical properties is worth considering in predicting corneal deformation after a removal of anterior corneal tissue.

## Background

Corneal refractive surgery corrects ocular refractive errors by removal of corneal tissue, consequently followed by corneal morphological change. The corneal biomechanical properties play an important role in maintaining the corneal normal morphology.

With the prolongation of time after corneal refractive surgery, there possibly have been problems such as refractive regression [1–3] and corneal ectasia [4–6]. Many investigators have paid their attentions to these problems, and believed there may have correlation with corneal biomechanical properties. The clinical follow-up results within one year after operation showed that the related parameters of corneal biomechanics will change [7–9], and the corneal biomechanical parameters fluctuate with time, such as corneal hysteresis (CH) and corneal resistance factor (CRF) output by Ocular Response Analyzer (ORA) [10–13]. The clinical observation results of dynamic corneal response parameters output by Corneal Visualization Scheimpflug Technology (Corvis ST) showed that stiffness parameter at applanation A1 (SPA1) decreased during 6 months after corneal refractive surgery [9, 14]. The above clinical retrospective studies showed that the changes of ORA and Corvis ST output parameters with time after corneal refractive surgery. However, the mechanical interpretation of these parameters is not clear, and these parameters cannot be directly used to predict the postoperative corneal morphology by using finite element method. Furthermore, most of the dynamic corneal response parameters are affected by central corneal thickness (CCT) [15, 16] and intraocular pressure (IOP) [17], there may be misunderstandings about the interpretation of the results after refractive surgery.

On the other hand, some animal experiments showed that the elastic modulus [18, 19] and the viscoelasticity [18] of cornea are different from those of control eyes after refractive surgery. However, the changes of corneal biomechanical properties are not clear with the time after refractive surgery. Based on this we speculate that the corneal biomechanical properties will change with time after refractive surgery.

As a biological tissue, the cornea could undergo a remolding process after corneal refractive surgery [20]. The corneal epithelial and corneal stromal cells will be adjusted [21–23] after refractive surgery, and the remodeling of corneal extracellular matrix is affected by the change of mechanical environment [24], and then affect the migration and metabolism of keratocytes. Some changes of corneal microstructure may cause the adjustment of corneal biomechanical properties. Furthermore, animal experiments showed that the transforming growth factor-β1 was still at a high level within 1 month after refractive surgery [25], it can promote the proliferation of corneal stromal cells [26]. Therefore, it is necessary to observe the biomechanical properties of cornea with time after refractive surgery.

The above clinical observation and animal experiment results show that the biomechanical properties of cornea will change after refractive surgery. Therefore, it is better to consider the changes of corneal biomechanical parameters in the preoperative design of refractive surgery and prediction of postoperative refraction. As we know, finite element method is often used to simulate and analyze the changes of corneal morphology and stress postoperatively [27, 28]. However, some finite element method simulations were performed by using preoperative or (partial) fixed corneal material parameters [27, 29, 30]. This may cause a large deviation between the simulation analysis results and the actual situation. Furthermore, because the change of corneal biomechanical properties with time after refractive surgery is not clear, the finite element method cannot better predict the corneal deformation at different times after refractive surgery, so the role of finite element method in the simulation design has not been brought into full play before refractive surgery. Therefore, to explore the biomechanical properties of cornea after refractive surgery plays an important role in the design of surgical scheme before refractive surgery.

One of the best ways to acquire corneal biomechanical property is uniaxial tests. Since it is impossible to apply uniaxial test for human cornea, and it is inconvenient and too expensive to perform corneal refractive surgery on animals by using the equipment for human, the aim to explore the changes of corneal biomechanical properties after refractive surgery go into a dilemma. Taking into account the common features of all types of corneal refractive surgery, removal of part of the anterior corneal tissue, we shall apply uniaxial tests to measure the biomechanical properties of the corneas after a removal of anterior corneal tissue in simulation to corneal refractive surgery in this study. The results will hope to improve our understanding on the variation of corneal biomechanical properties after corneal refractive surgery, and also to provide a mechanical basis for the occurrence of clinical phenomena such as iatrogenic keratectasia and refractive regression.

## Results

### In vivo measurement data

**Fig. 1:**
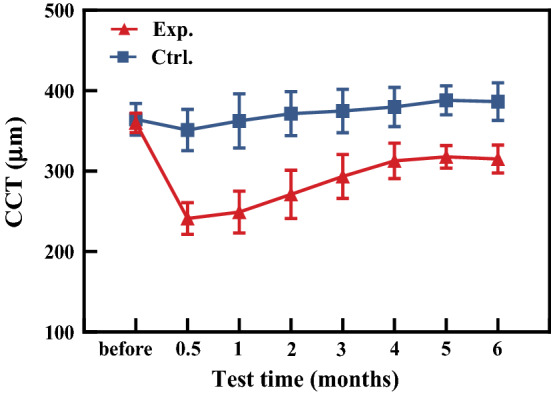
The changes of CCT with time. The triangle represents the experimental group and the square represents the control group

As shown in Fig. 1, CCT in experimental group decreased significantly after operation (*P* < 0.05). Within the 6 months, it increased firstly and then gradually stabilized. The ratios of CCT between the experimental and control groups were 0.68 at half of a month, 0.78 at the 3rd month, and 0.81 at the 6th month after operation.

**Fig. 2:**
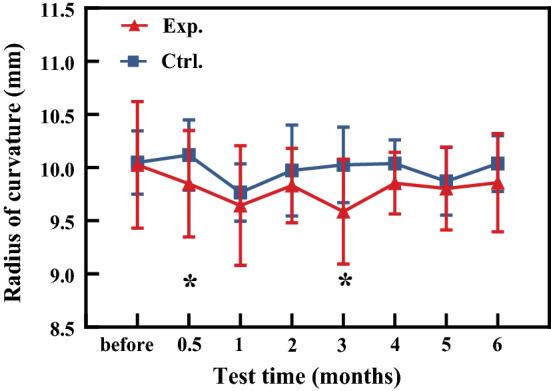
The changes of curvature radii of corneal posterior surface with time. The triangle represents the experimental group and the square represents the control group

Because the corneal anterior surface changes largely compared to posterior surface immediately after treatment, and the change of posterior surface is an important index for the diagnosis of corneal ectasia [31, 32] postoperatively, only the curvature radius of posterior surface were calculated based on OCT images according the method shown in our previous study [33]. Figure 2 shows that the curvature radius of corneal posterior surface in experimental group fluctuated slightly with time, and its mean value at all the time points was lower than its control group. There were statistical differences between experimental and control groups both at the half and third month after operation (*P < 0.05).

**Fig. 3:**
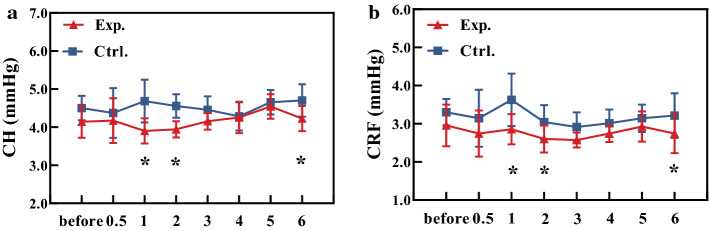
The changes of the CH (**a**) and CRF (**b**) with time. The triangle represents the experimental group and the square represents the control group

IOPcc, corneal compensated intraocular pressure, was no significantly different (*P* = 0.132) between experimental and control groups. (The IOPcc values of experimental and control groups were 16.13 ± 1.40 mmHg and 15.88 ± 1.16 mmHg, respectively.) CH and CRF fluctuated slightly with time, and there were statistically significant differences between experimental and control groups at the first, second, and sixth month after treatment (*P* < 0.05) (Fig. 3).

### Corneal elastics modulus

**Fig. 4:**
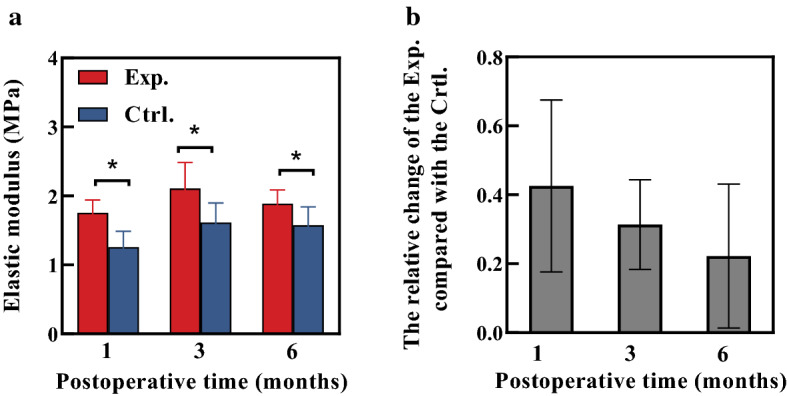
The changes of the elastic moduli with time (*P < 0.05) (**a**), the relative change of elastic modulus in experimental group compared with control group with postoperative time (**b**). Red and blue represent the experimental and control groups, respectively

The length and width of corneal strips were 7.96 ± 1.06 mm and 3.48 ± 0.17 mm, respectively. The elastic modulus in the physiological stress state is shown in Fig. 4a. Compared with the control group, the elastic modulus in experimental group increased significantly at each time points (*P* < 0.05). The change of corneal elastic modulus after treatment shows a trend of decreasing with time (Fig. 4b), although this trend has no statistical significance (*χ*^2^ = 1.800, *P* = 0.180).

### Stress relaxation data

**Fig. 5:**
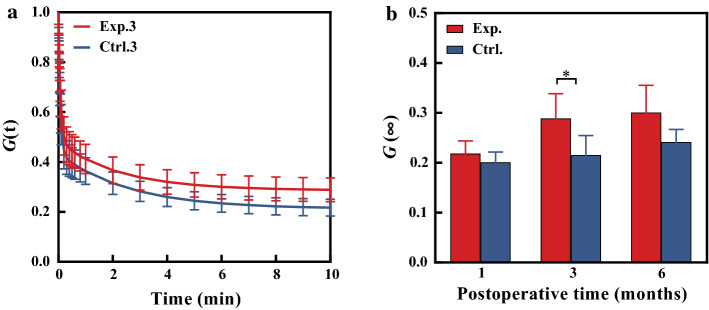
Stress-relaxation curves of corneal strips (**a**), the red and blue solid lines represent the experimental and control groups, respectively; the changes of stress relaxation degree with time (*P < 0.05) (**b**), red and blue represent the experimental and control groups, respectively

Figure 5a shows the stress relaxation curve at month 3 after treatment, the stress relaxation curves of the Exp.3 was above of the Ctrl.3. Mean values of stress relaxation limit *G*(∞) of experimental group at three time points were smaller than those of the control group. The difference was significant at the 3rd month after treatment (Fig. 5b).

### Corneal collagen fibrils data

**Fig. 6:**
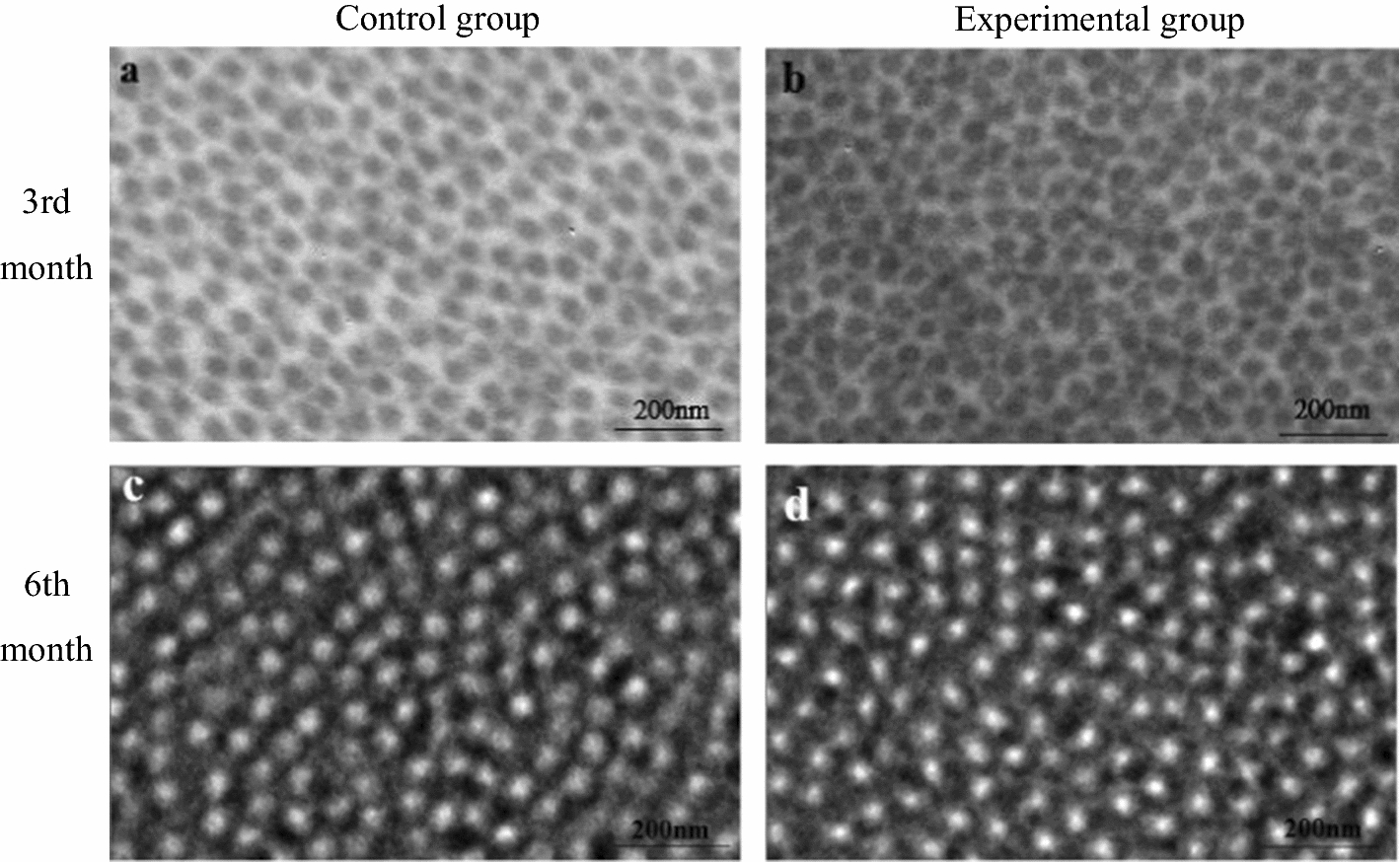
TEM images (×5000) of corneal collagen fibrils

**Fig. 7:**
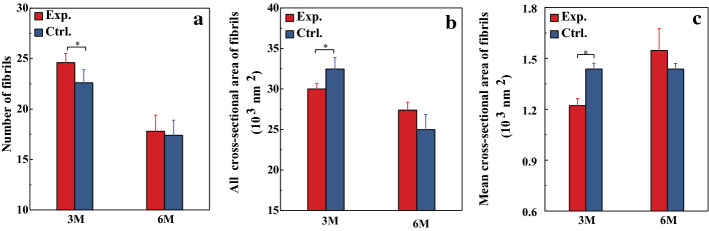
Measurement parameters of collagen fibrils, red and blue represent the experimental and control groups, respectively

Figure 6 shows the TEM images of corneal specimen (The  results  of TEM at the 1st month after operation were failed). We randomly selected 5 regions with the same size (0.302 × 0.270 μm^2^) on the TEM images and counted the total number, the cross-sectional area of collagen fibrils and mean cross-sectional area of one fibril (Fig. 7). At the 3rd month after treatment, the total number of collagen fibrils in experimental group was greater than that of control group, while the total cross-sectional area of collagen fibrils and the mean cross-sectional area per collagen fibril did the opposite (all *P* < 0.05, Fig. 7). At the 6th month after treatment, there were no significant differences between experimental and control groups (all *P* > 0.05, Fig. 7).

### Corneal deformation and stress distribution predicted by FEM

**Fig. 8:**
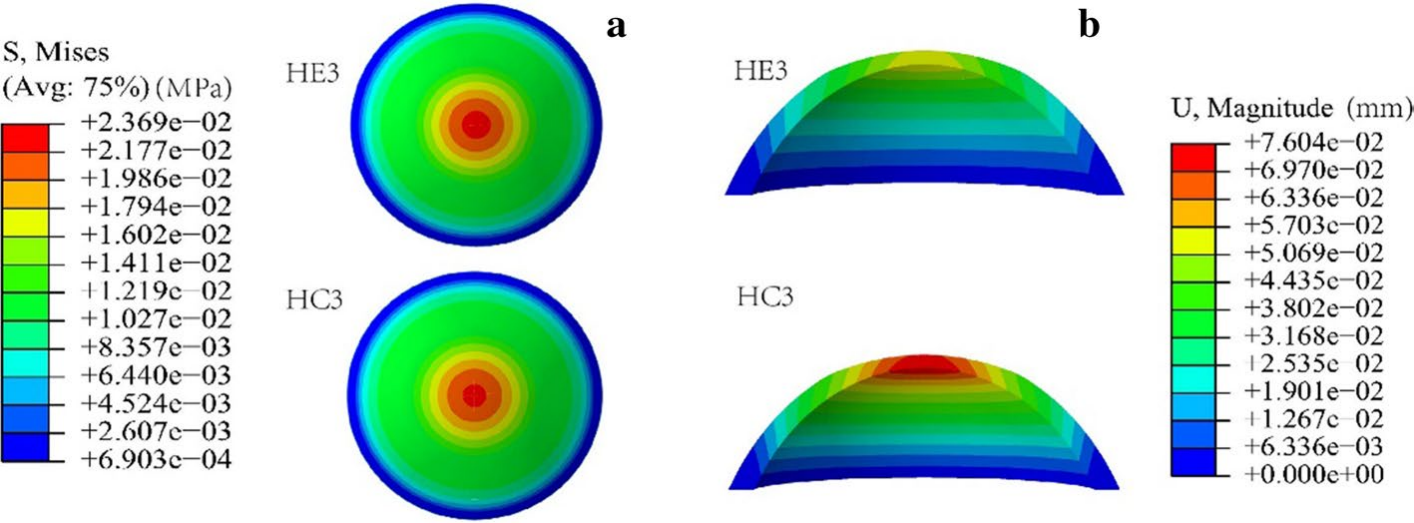
Stress (**a**) and displacement (**b**) distribution

Figure 8 shows the stress and displacement distribution of HE3 and HC3, the finite element model established with the elastic modulus of the Exp. and Ctrl. groups when the IOP was 15 mmHg. It could be seen that the stress and displacement in the central area of cornea are greater than those in the other region (Fig. 8). Both the corneal apex displacement (0.052 mm) and refractive power (~ 32.70 D) of HE3 were smaller those (0.071 mm) and (~ 32.95 D) of HC3, respectively. The maximum von-Mises stress (~ 22.94 kPa) of HE3 was greater than that (~ 22.61 kPa) of HC3.

**Fig. 9:**
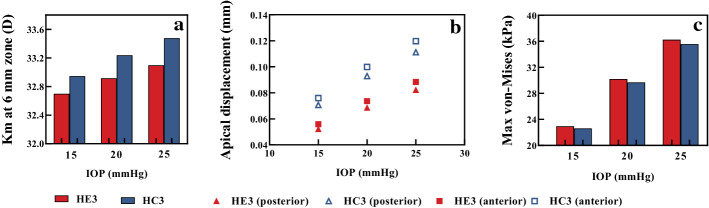
The variations of corneal morphology and stress of HE3 and HC3, the finite element model established with the elastic modulus of the Exp. and Ctrl. groups. **a** The corneal refractive power. **b** The apical displacement of corneal surface. **c** Maximum von Mises stress

When IOP changed, the calculated corneal refractive power and the corneal apical displacement in HE3 were smaller than those in HC3 slightly (Fig. 9a, b), and the maximum von-Mises stress in HE3 was greater than that in HC3 slightly (Fig. 9c). Under 25 mmHg, the differences of corneal refractive power and posterior surface apical displacement were about 0.4 D and 0.03 mm, respectively.

## Discussion

Based on the 6-month follow-up observations of rabbits after operation, including in vivo and in vitro experimental data, we explored the changes of corneal morphology, and mechanical parameters with postoperative time. Our results show that postoperative corneas undergo dynamic changes in corneal morphology and biomechanics. From the view of biomechanics, the first 3 months after operation is a critical period.

The results in this study are of great significance for the preoperative prediction and postoperative observation of human corneal refractive surgery, although we performed a non-standard corneal refractive surgery. In clinical corneal refractive surgery, the thickness of remained corneal stroma is not < 50% of the original corneal thickness. The researches have shown that the main risk factors of postoperative refractive regression and iatrogenic keratectasia are corneal thinning and changes of corneal biomechanical properties. Similarly, the amount of the removed anterior corneal tissue is not more than 50% in the animal model. Further, the location and amount of the removed corneal tissue are similar to those of corneal refractive surgery. In addition, the overcorrection or undercorrection after refractive surgery in clinic may be related to insufficient understanding of corneal biomechanical properties pre- and post-operatively. In fact, the results shown in Fig. 9 indicate that the changes of corneal morphology caused by different corneal biomechanical properties are different, which may affect the accuracy of postoperative refractive effect.

The results both from the uniaxial tests of corneal strips and ORA tests demonstrate that the corneal biomechanical properties after operation did undergo a changing process. From the morphological results and CH/CRF values shown in Figs. 2 and 3, we believe that the curvature radii of corneal posterior surface and CH/CRF were unstable within 3 months after operation. This point also can be deduced from the reported results, such as, the fluctuations of the corneal posterior surface [34–36], CH/CRF [10, 11] with time after operation. The elastic moduli of experimental group increased significantly at first, third and sixth month after operation, which was similar to the pervious results 1 month after operation [18, 19]. Furthermore, our results show that the stress relaxation of experimental group becomes slowed down compared with the control group at the 3rd month after operation (*P* < 0.05, Fig. 5).

The results of fiber observation showed that the fibrils will make an adjustment compared their control group at the 3rd month after operation (*P* < 0.05, Fig. 7). Corneal collagen fibrils were the load-bearing microstructures of the cornea. The corneal biomechanical properties are affected by the status of collagen fibrils [37–39]. Our results showed that the density of collagen fibrils increased, and the fibrils became thinner compared their control group at the 3rd month after operation (*P* < 0.05, Fig. 7). These changes were not found at the 6th month after operation (*P* > 0.05). This indicates that cornea tissue undergoes a remodeling process, which might lead to the fluctuation of the elastic modulus of the physiological range in a period of time.

Furthermore, from the changes of corneal biomechanical properties and collagen fibrils status, we suggest that close attentions to follow-up observations of the morphological and biomechanical changes of the cornea should be paid within 3 months after operation, which is helpful to find the early keratoconus. It agreed with that 25% of patients with corneal ectasia occurred within 3 months after corneal refractive surgery [40].

The numerical results suggest that the changes in corneal biomechanical properties should be considered in prediction of corneal postoperative morphology. The preoperative prediction of corneal morphology is an important aspect in surgical design. The corneal refractive outcome is related to the residual bed thickness of the cornea in the operation design, the removed actual thickness of corneal stroma in the operation and the corneal biomechanical properties [30, 41, 42]. In the present study, we only consider the difference of the elastic modulus between experiment group and control group, and the computation results showed the difference of corneal refractive power. If we had a fully understanding of the corneal biomechanical properties in different periods before and after operation, then we can get a good prediction of the postoperative refractive power based on the FEM, and then we may give a solution to the problem of postoperative overcorrection or undercorrection through comprehensive consideration of postoperative corneal biomechanical properties, residual bed thickness of the cornea, and the removed actual thickness of the stroma. Furthermore, we noticed that the displacement of posterior corneal surface, an important index after corneal refractive surgery [27], of HE3 was about 0.74 times that of HC3 (Fig. 9b) when IOP was 15 mmHg. It resulted from the changes of corneal biomechanical properties.

The limitation of this study was small sample number. Further studies are needed, including more samples and longer observation time to confirm these observations. Besides, this study did not concern the nonlinearity and anisotropy of corneal biomechanical properties. In the future, we shall explore the effects of nonlinear and anisotropic mechanical properties of cornea on postoperative corneal morphology based on fully understandings of corneal biomechanical properties after corneal refractive surgery.

## Conclusions

In short, after operation, the corneal biomechanical properties vary accordingly. With the postoperative time, the increase of corneal elastic modulus decrease gradually, and the difference of stress relaxation degree between experiment and control groups underwent a process from increase to decrease. Three months after operation is a critical period and the changes of the corneal biomechanical properties need to be observed for a long time. The variation of corneal biomechanical properties is worth considering in predicting corneal deformation after operation.

## Materials and methods

### The treatment of animal corneas

Twenty-four adult New Zealand white rabbits, healthy and free of ocular disease (All rabbit eyes were observed by slit lamp to exclude anterior segment lesions), were selected from the Laboratory Animal Center of Capital medical University. Animals were randomly divided into 3 groups, 8 rabbits for each group. (The weight of rabbits was measured, numbered 1, 2, …, 24 according to the weight from small to large, and then the number divided by 3. Those with the remainder of 1 were classified as group 1, those with the remainder of 2 were classified as group 2, and those with the remainder of 0 were classified as group 3.) The left eyes were treated as experimental (Exp.) group and the right eyes were kept as the self-control (Ctrl.) group. And the animals were raised up to 1, 3, or 6 months after treatment, respectively. The institutional review board of Capital Medical University approved this study (No. AEEI-2014-066), and all procedures adhered to the animal welfare.

In order to simulate corneal refractive surgery, corneal tissue (from corneal epithelial layer to the stroma: 6 mm in diameter and 30–50% of the original corneal thickness) was removed with a mechanical microkeratome (KM-5000, Wuxi Kangning medical apparatus Co. Ltd., China). After treatment, 0.3% tobramycin dexamethasone eye drops 4 times a day was applied to all experimental eyes within first week, and reduced to 2 times a day till 1 month after treatment.

### In vivo tests

In vivo tests were performed by Pachymeter SP3000 (TOMEY, Japan), ORA (Reichert Inc., USA), and optical coherence tomography (OCT, TOPCON, Mark II, Japan) before operation, at the 2nd, 4th week, and once every 4 weeks for 24 weeks. The measured parameters were central corneal thickness (CCT), corneal compensated intraocular pressure (IOPcc), CH/CRF and the corneal cross section images of the rabbit eyes.

### In vitro mechanical test

#### Preparation of corneal strip samples

Rabbits were anesthetized to death through intravenous injection of 25% urethane agent via rabbit auricular vein. Using a self-made double-edged knife cut along corneal superior–inferior direction. Corneal strip width was measured with a Vernier caliper. Its thickness was taken as CCT of the same cornea. The corneal strip samples were denoted by Exp. 1 (Exp. 3, Exp. 6) according to the experimental eyes from the animals that were executed at the first (third and sixth) month after operation.

#### Uniaxial tensile test

Corneal biomechanical properties were determined by uniaxial tensile test (Care-IBTC-50, Tianjin, China) at room temperature. To prevent dehydration during the test, corneal strips were kept moist with saline bath. After preload the hysteresis loop was stable, the stress–strain test at a tensile with a speed of 0.02 mm/s until them became 115% of the original length. After a 5-min recovery, all corneal strips were stretched with a speed of 0.5 mm/s as the same length of stress–strain test, stress-relaxation test were performed with 10 min.

#### Corneal elastic modulus and relaxation function

The stress–strain curve was linear in the range of load 0.025–0.05 N, corresponding to IOP in the corneal physiologic state [19]. The stress $\sigma =F / {A}_{0}$, where *A*_0_ is the initial cross-sectional area at the center of the corneal strip,* F* is the load. Assume the corneal strip was regular strip specimen. The strain $\varepsilon =\Delta l / {l}_{0}$, *l*_0_ is the length of the specimen, about the diameter of corneal cutting region, *∆l* is the displacement. Since the stress–strain relationship of corneal strip was regarded as linear in the corneal physiological range, the corneal elastic modulus was the ratio of stress to strain of the strip.

The following 2-term Prony model was chosen to fit stress relaxation data:

$$G (t )=1-{A}_{1}\left(1-{e}^{-\frac{t}{{\tau }_{1}}}\right)-{A}_{2}\left(1-{e}^{-\frac{t}{{\tau }_{2}}}\right),$$

where $G(t)=\sigma (t)/{\sigma }_{0}$ is the normalized stress-relaxation function, $\sigma \left(t\right)$ is the stress, ${\sigma }_{0}$ is the initial stress, *A*_1_, *A*_2_, *τ*_1_ and *τ*_2_ are stress relaxation parameters.

Stress relaxation limit *G*(∞) was defined by the normalized stress when the time was infinity, which was used to describe the stress relaxation degree, the larger value of *G*(∞) was, the smaller stress relaxation degree was.

### Collagen fibrils morphology observation

To observe the arrangement of collagen fibrils, one rabbit was randomly selected at the 1st, 3rd, and 6th month after operation, respectively. The corneal specimen, came from the corneal center area about 1 × 1 mm^2^, was fixed by 2.5% glutaraldehyde solution and was kept at 4 °C for 4 h. The morphology of collagen fibrils was observed by transmission electron microscopy (TEM, H7650, Hitachi, Japan), and the total number and the cross-sectional area of collagen fibrils were counted by software of Image Pro Plus (V6.0, Media Cybernetics, USA).

### Corneal finite element model

In order to know the differences of corneal morphology due to the changes of corneal biomechanical properties after corneal refractive surgery, we applied finite element model (FEM) method to analyze the data of the corneal diopters and the apical displacement of corneal posterior surface. We established an axisymmetric healthy corneal geometric model, where the curvature radius of anterior and posterior corneal surface were 7.8 mm and 6.8 mm, respectively, the CCT was 0.55 mm, transverse diameter of cornea was 12 mm. The corneal geometric model after corneal refractive surgery was constructed on the basis of the normal human corneal geometric model. The amount of corneal tissue removed was 30% of the CCT, and the diameter of optical zone was 6.5 mm. Cornea was regarded as a homogeneous, incompressible material. The corneal elastic modulus of experimental and control eyes at month 3 after treatment were applied, where the FEM were denoted by HE3 and HC3, respectively.

Corneal geometric model under unloaded state was obtained by the methods in references [43]. In Abaqus (V6.13, RI, USA), the models were fixed completely at the boundary. Local refinement of the mesh along depth direction of the cornea was applied. To predict the effect of IOP fluctuation on corneal deformation, the uniform forces of 15 mmHg, 20 mmHg, 25 mmHg were applied on corneal inner surface, represented IOP values.

According to the results of finite element simulation for each model, the corneal refractive power was calculated in the range of 3 mm from the corneal apex, the corneal refractive power was expressed by *K*_m_.

$${K}_{\mathrm{m}}={K}_{\mathrm{a}}+{K}_{\mathrm{p}}-\frac{t}{{n}_{2}}{K}_{\mathrm{a}}{K}_{\mathrm{p}},$$

$${K}_{\mathrm{a}}=\frac{{n}_{2}-{n}_{1}}{{r}_{1}}, {K}_{\mathrm{p}}=\frac{{n}_{3}-{n}_{2}}{{r}_{2}},$$

where *K*_a_ and *K*_p_ are the refractive power of the anterior and posterior corneal surfaces, *r*_1_ and *r*_2_ are the radius of curvature of the anterior and posterior cornea, respectively, *n*_2_, *n*_1_ and *n*_3_ represent the refractive index of the cornea, air and aqueous humor, *t* is the CCT.

**Fig. 10:**
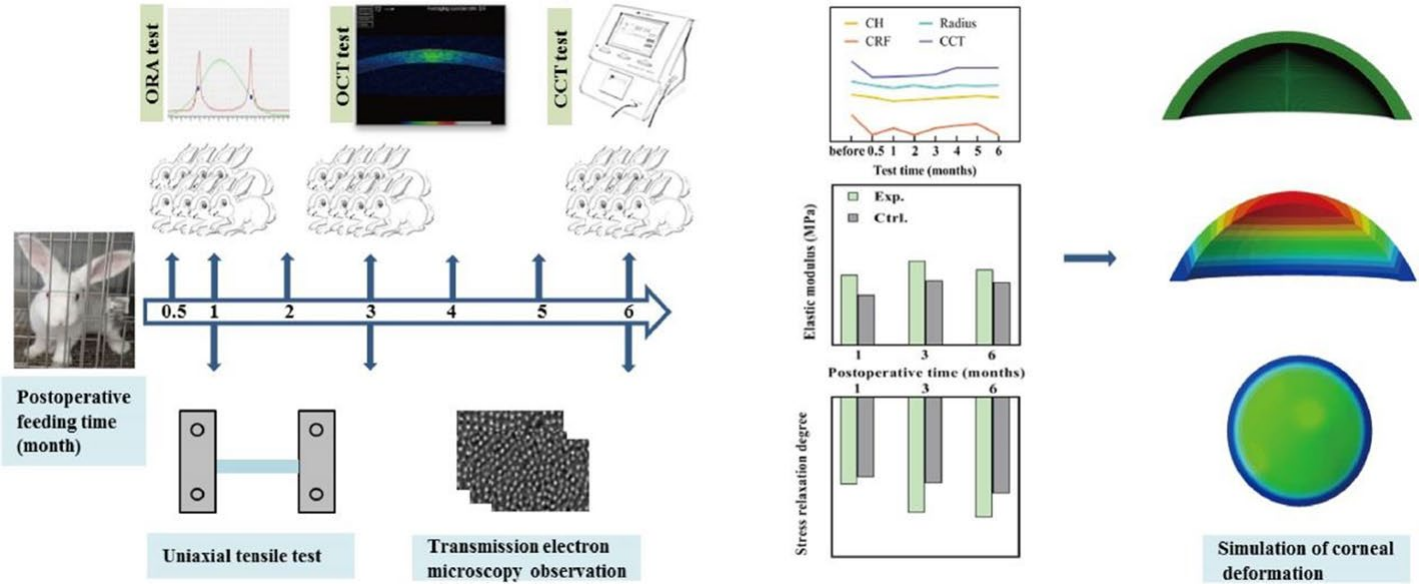
Schematic diagram of the experimental setup

The schematic diagram of the experimental setup is shown in Fig. 10.

### Statistical analysis

Statistical analysis was performed using SPSS (V21.0, IBM, USA). Data were tested for normality using Shapiro–Wilk test, and data were expressed as mean ± SD or median (inter-quartile range, IQR). The morphological parameters and in vivo corneal biomechanical data of different groups were compared by repeated-measures analysis of variance or Friedman test, and paired *t* test or Wilcoxon signed rank test was used to compare the data of uniaxial test and the morphological parameters of collagen fibrils of different groups. The level of statistical significance was set to *P* < 0.05.

## Data Availability

The data used to support the findings of this study are available from the corresponding author upon request.

## Abbreviations

CRS: Corneal refractive surgery
CCT: Central corneal thickness
CH: Corneal hysteresis
CRF: Corneal resistance factor
ORA: Ocular response analyzer
Corvis ST: Corneal visualization Scheimpflug technology
IOP: Intraocular pressure
FEM: Finite element model
OCT: Optical coherence tomography
IOPcc: Corneal compensated intraocular pressure
TEM: Transmission electron microscopy

## References

[1] Alio JL, Soria FA, Abbouda A, Peña-García P (2016). Fifteen years follow-up of photorefractive keratectomy up to 10 D of myopia: outcomes and analysis of the refractive regression. Br J Ophthalmol.

[2] Ivarsen A, Hjortdal J (2012). Seven-year changes in corneal power and aberrations after PRK or LASIK. Invest Ophthalmol Vis Sci.

[3] Blum M, Lauer AS, Kunert KS, Sekundo W (2019). 10-year results of small incision lenticule extraction. J Refract Surg.

[4] Wang Y, Cui C, Li Z, Tao X, Zhang C, Zhang X (2015). Corneal ectasia 6.5 months after small-incision lenticule extraction. J Cataract Refract Surg.

[5] Rad AS, Jabbarvand M, Saifi N (2004). Progressive keratectasia after laser in situ keratomileusis. J Refract Surg.

[6] Twa MD, Nichols JJ, Joslin CE, Kollbaum PS, Edrington TB, Bullimore MA (2004). Characteristics of corneal ectasia after LASIK for myopia. Cornea.

[7] Wu Z, Wang Y, Zhang J, Chan TCY, Ng ALK, Cheng GPM (2017). Comparison of corneal biomechanics after microincision lenticule extraction and small incision lenticule extraction. Br J Ophthalmol.

[8] Cao K, Liu L, Yu T, Chen F, Bai J, Liu T (2020). Changes in corneal biomechanics during small-incision lenticule extraction (SMILE) and femtosecond-assisted laser in situ keratomileusis (FS-LASIK). Lasers Med Sci.

[9] Wu D, Liu C, Li B, Wang D, Fang X (2020). Influence of cap thickness on corneal curvature and corneal biomechanics after SMILE: a prospective, contralateral eye study. J Refract Surg.

[10] Wu D, Wang Y, Zhang L, Wei S, Tang X (2014). Corneal biomechanical effects: small-incision lenticule extraction versus femtosecond laser-assisted laser in situ keratomileusis. J Cataract Refract Surg.

[11] Chen M, Yu M, Dai J (2016). Comparison of biomechanical effects of small incision lenticule extraction and laser-assisted subepithelial keratomileusis. Acta Ophthalmol.

[12] Ryan DS, Coe CD, Howard RS, Edwards JD, Bower KS (2011). Corneal biomechanics following epi-LASIK. J Refract Surg.

[13] Wang B, Zhang Z, Naidu RK, Chu R, Dai J, Qu X (2016). Comparison of the change in posterior corneal elevation and corneal biomechanical parameters after small incision lenticule extraction and femtosecond laser-assisted LASIK for high myopia correction. Cont Lens Anterior Eye.

[14] Lee H, Roberts CJ, Kim TI, Ambrosio R, Elsheikh A, Yong Kang DS (2017). Changes in biomechanically corrected intraocular pressure and dynamic corneal response parameters before and after transepithelial photorefractive keratectomy and femtosecond laser-assisted laser in situ keratomileusis. J Cataract Refract Surg.

[15] Miki A, Maeda N, Ikuno Y, Asai T, Hara C, Nishida K (2017). Factors associated with corneal deformation responses measured with a dynamic scheimpflug analyzer. Invest Ophthalmol Vis Sci.

[16] Lee H, Kang DSY, Ha BJ, Choi JY, Kim EK, Seo KY (2018). Biomechanical properties of the cornea using a dynamic scheimpflug analyzer in healthy eyes. Yonsei med J.

[17] Wang W, He M, He H, Zhang C, Jin H, Zhong X (2017). Corneal biomechanical metrics of healthy Chinese adults using Corvis ST. Cont Lens Anterior Eye.

[18] Zhang H, Khan MA, Zhang D, Qin X, Lin D, Li L (2018). Corneal biomechanical properties after FS-LASIK with residual bed thickness less than 50% of the original corneal thickness. J Ophthalmol.

[19] Wang X, Li X, Chen W, He R, Gao Z, Feng P (2017). Effects of ablation depth and repair time on the corneal elastic modulus after laser in situ keratomileusis. Biomed Eng Online.

[20] Kivanany PB, Grose KC, Tippani M, Su S, Petroll WM (2018). Assessment of corneal stromal remodeling and regeneration after photorefractive keratectomy. Sci Rep.

[21] Luft N, Ring MH, Dirisamer M, Mursch-Edlmayr AS, Kreutzer TC, Pretzl J (2016). Corneal epithelial remodeling induced by small incision lenticule extraction (SMILE). Invest Ophthalmol Vis Sci.

[22] Romito N, Trinh L, Goemaere I, Borderie V, Laroche L, Bouheraoua N (2020). Corneal remodeling after myopic SMILE: an optical coherence tomography and in vivo confocal microscopy study. J Refract Surg.

[23] Dong Z, Zhou X, Wu J, Zhang Z, Li T, Zhou Z (2014). Small incision lenticule extraction (SMILE) and femtosecond laser LASIK: comparison of corneal wound healing and inflammation. Br J Ophthalmol.

[24] Du GL, Chen WY, Li XN, He R, Feng PF (2017). Induction of MMP-1 and-3 by cyclical mechanical stretch is mediated by IL-6 in cultured fibroblasts of keratoconus. Mol Med Rep.

[25] Liu L, Cheng W, Wu D, Chen L, Yu S, Zuo T (2020). The differential expression of cytokines and growth factors after SMILE compared with FS-LASIK in rabbits. Invest Ophthalmol Vis Sci.

[26] Ljubimov AV, Saghizadeh M (2015). Progress in corneal wound healing. Prog Retin Eye Res.

[27] Fang L, Wang Y, Yang R, Deng S, Deng J, Wan L (2020). Effects of the LASIK flap thickness on corneal biomechanical behavior: a finite element analysis. BMC Ophthalmol.

[28] Sinha Roy A, Dupps WJ, Roberts CJ (2014). Comparison of biomechanical effects of small-incision lenticule extraction and laser in situ keratomileusis: finite-element analysis. J Cataract Refract Surg.

[29] Vahdati A, Seven I, Mysore N, Randleman JB, Dupps WJ (2016). Computational biomechanical analysis of asymmetric ectasia risk in unilateral post-LASIK ectasia. J Refract Surg.

[30] Fang L, Ma W, Wang Y, Dai Y, Fang Z (2020). Theoretical analysis of wave-front aberrations induced from conventional laser refractive surgery in a biomechanical finite element model. Invest Ophthalmol Vis Sci.

[31] Fan R, Chan TC, Prakash G, Jhanji V (2018). Applications of corneal topography and tomography: a review. Clin Exp Ophthalmol.

[32] Ganesh S, Patel U, Brar S (2015). Posterior corneal curvature changes following refractive small incision lenticule extraction. Clin Ophthalmol.

[33] Zhang H, Xiao Q, Cao X, Zhang D, Li L (2017). Age-related variations of rabbit corneal geometrical and clinical biomechanical parameters. Biomed Res Int.

[34] Yu M, Chen M, Dai J (2019). Comparison of the posterior corneal elevation and biomechanics after SMILE and LASEK for myopia: a short- and long-term observation. Graefes Arch Clin Exp Ophthalmol.

[35] Bao F, Cao S, Wang J, Wang Y, Huang W, Zhu R (2019). Regional changes in corneal shape over a 6-month follow-up after femtosecond-assisted LASIK. J Cataract Refract Surg.

[36] Chan TC, Liu D, Yu M, Jhanji V (2015). Longitudinal evaluation of posterior corneal elevation after laser refractive surgery using swept-source optical coherence tomography. Ophthalmology.

[37] Liu X, Wang L, Ji J, Yao W, Wei W, Fan J (2014). A mechanical model of the cornea considering the crimping morphology of collagen fibrils. Invest Ophthalmol Vis Sci.

[38] Wu Q, Applegate BE, Yeh AT (2011). Cornea microstructure and mechanical responses measured with nonlinear optical and optical coherence microscopy using sub-10-fs pulses. Biomed Opt Express.

[39] Liu T, Shen M, Huang L, Xiang Y, Li H, Zhang Y (2020). Characterization of hyperelastic mechanical properties for youth corneal anterior central stroma based on collagen fibril crimping constitutive model. J Mech Behav Biomed Mater.

[40] Randleman JB, Woodward M, Lynn MJ, Stulting RD (2008). Risk assessment for ectasia after corneal refractive surgery. Ophthalmology.

[41] Kling S, Hafezi F (2017). Corneal biomechanics—a review. Ophthalmic Physiol Opt.

[42] Wang M, Zhang Y, Wu W, Young JA, Hatch KM, Pineda R (2018). Predicting refractive outcome of small incision lenticule extraction for myopia using corneal properties. Transl Vis Sci Technol.

[43] Nguyen BA, Roberts CJ, Reilly MA (2019). Biomechanical impact of the sclera on corneal deformation response to an air-puff: a finite-element study. Front Bioeng Biotechnol.

